# Conditionally Active, pH-Sensitive Immunoregulatory Antibodies Targeting VISTA and CTLA-4 Lead an Emerging Class of Cancer Therapeutics

**DOI:** 10.3390/antib12030055

**Published:** 2023-08-30

**Authors:** F. Donelson Smith, Robert H. Pierce, Thomas Thisted, Edward H. van der Horst

**Affiliations:** 1 Sensei Biotherapeutics, Inc., 1405 Research Blvd., Suite 125, Rockville, MD 20850, USA; tthisted@senseibio.com; 2 282088 US Highway 101, Port Townsend, WA 98368, USA; roberthamiltonpierce@gmail.com

**Keywords:** pH selective antibodies, Warburg effect, cancer immunotherapy, immune checkpoint inhibitors

## Abstract

Immune checkpoints and other immunoregulatory targets can be difficult to precisely target due to expression on non-tumor immune cells critical to maintaining immune homeostasis in healthy tissues. On-target/off-tumor binding of therapeutics results in significant pharmacokinetic and pharmacodynamic problems. Target-mediated drug disposition (TMDD) significantly limits effective intratumoral drug levels and adversely affects anti-tumor efficacy. Target engagement outside the tumor environment may lead to severe immune-related adverse events (irAEs), resulting in a narrowing of the therapeutic window, sub-optimal dosing, or cessation of drug development altogether. Overcoming these challenges has become tractable through recent advances in antibody engineering and screening approaches. Here, we review the discovery and development of conditionally active antibodies with minimal binding to target at physiologic pH but high-affinity target binding at the low pH of the tumor microenvironment by focusing on the discovery and improved properties of pH-dependent mAbs targeting two T cell checkpoints, VISTA and CTLA-4.

## 1. Introduction

Monoclonal antibodies (mAbs) have proven to be effective therapeutics across a wide array of diseases. Indeed, since the approval of the first therapeutic mAb by the FDA in 1986 (Othoclone OKT3) [1], over 100 additional mAbs have been approved, establishing antibodies and antibody-based therapeutics as a remarkably successful drug class [2]. Critical determinants of the success of mAbs as a therapeutic class are their exquisite selectivity coupled with their high affinity target antigen binding. Typically, mAb therapeutics have binding constants (K_D_) in the picomolar to low nanomolar range and no significant cross-reactivity to non-target proteins, even to closely related protein family members. Due to these features, mAbs are not only useful as ‘naked’ therapeutics themselves, but they also form the core targeting technology in other therapeutic modalities, such as CAR-Ts (chimeric antigen receptor T cells), ADCs (antibody-drug conjugates), and targeted radiopharmaceuticals.

Unlike small molecule therapeutics, where dose-dependent off-target toxicities are relatively commonplace, the toxicities associated with mAbs are overwhelmingly the result of “exaggerated pharmacology” from on-target activity in non-target tissues [3]. On-target/off-tumor activity is a particularly prevalent obstacle in immuno-oncology, given that prominent immunotherapy targets are expressed on subsets of normal immune cells that have important immunoregulatory functions in non-tumor tissues. Thus, efforts are intensifying to restrict the activity of antibody-based therapeutics to the tumor microenvironment [4,5,6,7,8,9].

Here, we will focus first on delineating the need for conditionally active antibodies that selectively bind their target antigen in the tumor microenvironment and then on recent advances in antibody engineering and screening, which have demonstrated proof-of-concept of this approach using pH as the environmental trigger. These initial efforts have opened up the possibility of effectively engaging previously “undruggable” targets or increasing the therapeutic index of drugs which are active but associated with a high frequency of severe toxicity linked to on-target/off-tumor activity.

## 2. VISTA and CTLA-4: Two Immune Checkpoints That Illustrate Distinct Hurdles to Overcome in Immuno-Oncology Drug Development

**Bypassing VISTA expression in normal tissues.** VISTA (V-domain Ig suppressor of T-cell activation/ B7-H5) is a B7 family member that is highly expressed on myeloid-lineage cells including dendritic cells, neutrophils, monocytes, macrophages, and myeloid-derived suppressor cells [10]. Expression of VISTA has also been described on certain subsets of T cells (e.g., Tregs, TIL, naïve CD4+ T cells) and occasionally on tumor cells [11]. Preclinical studies using VISTA knockout mice or anti-VISTA antibodies showed significant enhancement of antigen-specific T cell responses in vaccine models and anti-tumor activity in a variety of immunocompetent, syngeneic tumor models [12,13]. Despite these compelling preclinical studies, attempts to develop inhibitory antibody drugs against VISTA have been challenging due to the strong expression on neutrophils and monocytes. Binding to VISTA on these myeloid cells in the blood leads to rapid internalization and clearance from the blood through target-mediated drug disposition (TMDD), resulting in poor plasma residence time. Target-mediated drug disposition and the resultant low free drug concentration in plasma can severely limit biodistribution into the tumor, leading to inadequate target coverage in the TME. To date, all anti-VISTA antibodies that bind VISTA at physiologic pH in the blood have demonstrated non-linear drug elimination, indicating significant TMDD [14,15,16,17]. 

Janssen (J&J) performed the initial first-in-human, phase 1 clinical trial (NCT02671955) for an anti-VISTA mAb (JNJ-61610588) [18]. At subtherapeutic dose levels (0.3 mgs/kg IV), grade 3 cytokine release syndrome and encephalopathy were encountered, leading JNJ to stop the trial [18]. JNJ-61610588 utilized an Fc-competent IgG1 framework, which was chosen based on preclinical studies showing that Fc-incompetent frameworks were significantly inferior in terms of immunostimulatory and anti-tumor effects [19,20]. Although not formally demonstrated with JNJ-61610588, based on in vitro studies showing IgG1-dependent myeloid activation, it appears likely that the coating of myeloid cells in the blood with an IgG1 anti-VISTA mAb led to activation in circulation, resulting in cytokine release syndrome (CRS). Consistent with this observation, it has been reported that activation of myeloid cells (e.g., macrophages/monocytes, dendritic cells, etc.) and endothelial cells could lead to the release of pro-inflammatory cytokines (e.g., IL-6, IL-8, IL-10, TNF-α) by myeloid cells, which then could trigger T cell activation. This establishes a positive feedback loop wherein activated T cells produce IFN-γ and TNF-α, further activating myeloid cells, resulting in immune over-activation and CRS [21]. In support of this hypothesis, Kineta is developing an anti-VISTA IgG1 antibody engineered to reduce binding to FcRs and has demonstrated significant diminution of cytokine secretion in a preclinical CRS model [15]. Although decreased affinity for Fc receptors appears to have mitigated CRS risk, binding of KVA12123 to VISTA on blood cells at physiologic pH fails to eliminate significant TMDD [15], as this is a consequence of CDR-dependent, target-mediated internalization and clearance.

**Improving the therapeutic window of anti-CTLA-4 mAbs.** Ipilimumab (Ipi), an antagonist anti-CTLA-4 IgG1 mAb, was the first antibody immune checkpoint inhibitor (CPI), first approved by the FDA in 2011 for the treatment of melanoma [22]. Ipi, either alone or in combination with nivolumab (Nivo, anti-PD-1), is currently approved in the treatment of multiple tumor types [23,24] despite the incidence of severe immune-mediated adverse events, which most frequently affect the gastrointestinal, skin, and endocrine systems [25]. In contrast to inhibitory PD-1 mAbs, ipilimumab’s toxicity is dose-dependent, and the dose-limiting toxicities (DLTs) prevent dose escalation to achieve maximal anti-tumor effects. Combination treatment with ipilimumab and PD-1 blockade does increase anti-tumor activity, but the increased efficacy of the combination treatment correlates with increased incidence and severity of irAEs [26,27]. 

CTLA-4 is expressed on T cells in the early priming phase of an immune response, functioning to suppress excessive T cell activation through sequestration of CD80/86 that would otherwise provide co-stimulatory “signal 2” by engaging CD28 [28]. CTLA-4 is also highly expressed on Treg populations. Tregs appear in both tumors and in healthy tissues, where they normally play a critical role in maintaining immune homeostasis [29]. Loss of CTLA-4 in Tregs has been shown to perturb their normal development and suppressive function, which may lead to autoimmune sequelae [30]. Recent data demonstrate that Fc γR-mediated antibody-dependent cellular cytotoxicity (ADCC) is a critical, and perhaps dominant, mechanism underlying anti-CTLA-4 activity in the tumor microenvironment [31,32]. Anti-CTLA-4 antibodies with reduced ADCC function (IgG2, tremelimumab) have decreased activity [33,34], whereas afucosylated Fc domains with increased ADCC demonstrate enhanced anti-tumor activity [35]. The density of intratumoral CTLA-4 on the cell surface of Tregs is greater than that of peripheral and tissue-resident Tregs, which may provide some selective activity within the tumor, but it remains unclear whether ADCC also contributes to loss of Treg-mediated immune homeostasis in non-tumor tissue. The leading hypothesis is that CTLA-4 activity on Tregs is essential for the development of functional, immunosuppressive Tregs and that inhibition or loss of CTLA-4 leads to Treg dysfunction and auto-immune-like tissue pathology [36]. 

In the case of both anti-VISTA mAbs and anti-CTLA-4 mAbs, the fundamental challenges to drug development include widespread expression of the target proteins in non-tumor tissue, and subsequently, the inability of conventional antibodies to discriminate between target in the TME versus non-tumor tissue. The principal obstacle for anti-VISTA antibodies has been on-target/off-tumor binding to myeloid cells in the blood, resulting in high potential for CRS, whereas for targeting CTLA-4, the issue lies in disrupting the balance of Treg function and inducing autoimmune syndromes. The desire to increase the therapeutic window of inhibitory mAbs by limiting on-target/off-tumor activity has led investigators to pursue the development of antibodies that are selectively active in the tumor microenvironment. We now consider current approaches to address these critical issues.

## 3. Harnessing Unique Features of the TME for Conditional mAb Activity

The pathological growth of malignant cells results in changes within the tumor microenvironment that differ significantly from normal tissue in a variety of ways. Altered metabolic activity and unconstrained growth, coupled with increased cell death, leads to hypoxia, increased expression and activation of proteases, high lactate/low pH, high extracellular ATP levels, REDOX imbalance, and high levels of cell-free DNA [37,38]. Several of these TME characteristics have been successfully leveraged to enable tumor-selective “conditional activation” of mAbs. For example, protease-mediated activation of antibodies through cleavage of an inhibitory peptide domain has been utilized to activate antibody prodrugs (so-called “probodies”) [39,40,41,42]. However, the tumor selectivity of this approach may be compromised by the fact that the cleavage—and, hence, activation—is an irreversible step and may result in re-circulation of activated antibody out of the TME and into systemic circulation [41]. Conversely, incomplete removal of the ‘shielding’ peptide domain would leave the ‘probody’ in an inert state.

An alternative approach involves capitalizing on the unique biochemical features of the TME to induce reversible activation of mAb binding. While some progress has been made with developing conditionally active antibodies sensitive to high extracellular ATP levels [43,44], we will focus on the significant advances made by multiple research groups towards developing pH-sensitive binders, which preferentially engage their target antigens in the acidic TME.

**Leveraging the Warburg Effect for pH-dependent mAb Activity.** Roughly 100 years ago, Otto Warburg reported that cancer utilizes aerobic fermentative glycolysis in preference to cellular respiration (aka oxidative phosphorylation; OXPHOS), in contrast to normal cells [38,45,46,47,48]. This phenomenon—commonly referred to as the Warburg effect—was surprising because aerobic glycolysis is relatively inefficient in terms of ATP production as compared to OXPHOS metabolism: per glucose molecule, the ratio of ATP production is 1 versus 18, respectively. Interestingly, this metabolic preference for glycolysis appears to be a hallmark not just of cancer but of rapidly proliferating cells in general, including yeast and T cells, because glycolysis produces the anabolic building blocks required to support proliferation. The shift from OXPHOS to aerobic glycolysis requires the induction of multiple glycolytic enzymes that catalyze the conversion of glucose to pyruvate and, ultimately, lactic acid. Expression of these critical metabolic enzymes is commonly orchestrated through the myc pathway and/or HIF-1 [49,50]. Interestingly, many viruses hijack these pathways early in the infectious process to drive cellular Warburg metabolism to secure sufficient substrate for rapid replication [51]. The overproduction of lactate and extrusion of lactic acid into the interstitia reverses the normal tissue pH gradient such that extracellular pH is acidified (~pH 6) [52]. In the case of T cells, activation leads to a metabolic shift from OXPHOS in non-proliferative T cells to aerobic glycolysis during T cell expansion, explaining the recent data demonstrating the existence of microniches of low pH in activated lymph nodes [53]. In tumors, aerobic glycolysis in multiple cell types drives the production of lactic acid, which is exported by a family of monocarboxylate transporters (MCTs), resulting in the low extracellular pH (range ~6–6.8) [54,55,56,57,58,59,60,61,62,63]. Thus, tumors likely display complex pH gradients that are shaped by local hypoxia, necrosis, vascularization, and metabolic activity [64]. 

**Figure 1:**
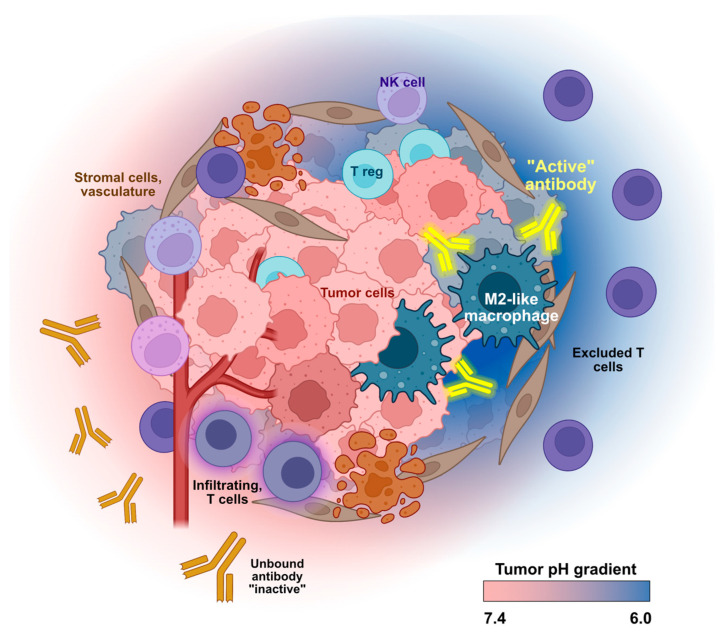
Conditional activity of pH-selective therapeutic antibodies. Altered tumor metabolism results in an acidic extracellular tumor microenvironment. Antibodies with pH-dependent binding act at the site of the tumor (yellow “active” antibodies) and will not engage their target at normal physiological pH (orange “inactive” antibodies). This low pH-selective binding will reduce on-target/off-tumor side effects and improve safety and pharmacokinetic properties. Created in part with BioRender.com.

Initial attempts to engineer pH sensitivity into antibodies aimed to exploit pH to regulate antibody-antigen or antibody-FcR interactions during the processes of uptake and endocytic recycling [65,66]. This strategy has proven to be successful, particularly for improving antibody pharmacokinetics; however, it is focused on intracellular (endosomal) compartment pH and is less concerned with target engagement in the extracellular space [9,67]. Here, we focus on the opposite design goal: development of therapeutic antibodies that leverage reversible, pH-selective binding in the acidic tumor microenvironment to mitigate on-target/off-tumor activity and widen the therapeutic window (Figure 1). There is a clear therapeutic case for the development of conditionally active, pH-dependent antibodies. Here, we review examples of pH-selective inhibitory antibodies targeting the VISTA and CTLA-4 checkpoints with a focus on the discovery and key properties of these biologics.

**VISTA—An Immune Checkpoint with Intrinsic pH-dependence.** In 2019, Alan Korman’s group at Bristol Myers Squibb published a landmark paper establishing that VISTA is a pH-dependent binder for P-selectin glycoprotein ligand-1 (PSGL-1) on T cells [68]. Importantly, PSGL-1 had previously been recognized as an inhibitory receptor expressed by T cells [69,70]. In the work by Johnson et al., [68] the authors describe how VISTA acts as a pH-sensing switch, constraining the activity of the VISTA/PSGL-1 checkpoint to low pH microenvironments due to a unique histidine-rich sequence in its PSGL-1 binding domain. Engagement of this VISTA/PSGL-1 checkpoint requires two simultaneous protein modifications. T cell activation induces the post-translational modification of PSGL-1 by tyrosine sulfation, and low pH-induced histidine protonation creates a positive charge shift on VISTA, converting it from an inactive binding state to an active one. These positively charged histidine residues interact with the negatively charged sulfate groups on PSGL-1 [68]. Binding to and activation of PSGL-1 on T cells leads to an inhibitory cascade involving inhibition of TCR signaling, upregulation of PD-1, and, ultimately, exhaustion [70]. Thus, the PSGL-1 (T cell)/VISTA (antigen-presenting cell) checkpoint leverages the Warburg effect associated with T cell activation and proliferation to limit over-exuberant T cell expansion [10,68]. 

The identification of the intrinsic “histidine switch” epitope in the extracellular domain of VISTA provided Johnston et al. [68] an opportunity to develop conditionally active, pH-sensitive mAbs that selectively bind the active (i.e., protonated) form of VISTA, blocking its interaction with PSGL-1 and circumventing the on-target/off-tumor binding to blood cells responsible for TMDD and CRS. After the initial identification of VISTA.4, an antibody which bound this histidine switch epitope, Johnson et al. engineered VISTA.16 (a pH-non-selective mAb) and its progeny, VISTA.18, a high-affinity, pH-dependent anti-VISTA mAb capable of blocking the interaction between PSGL-1 and the active, protonated VISTA at low pH [68].

The crystal structure of VISTA.18 Fab bound to VISTA’s immunoglobulin domain (IgV) revealed a critical interaction between two negatively charged amino acids (E100 and D102) in the antibody CDRs and two histidine residues (H153 and H154) in VISTA that appear to underlie the selectivity for binding at low pH. VISTA.18 showed no significant TMDD; a single-dose PK study in cynomolgus macaques demonstrated a mean blood residence time of 717 h, compared to 7.6 h for the parental antibody VISTA.4 (derived from the same epitope bin), which retains binding at neutral pH. Furthermore, using fluorescently labeled antibodies and in vivo imaging, VISTA.18 demonstrated preferential biodistribution into tumors in human VISTA knock-in mice, whereas VISTA.16 (no pH selectivity) was observed predominantly in leukocyte-rich organs (e.g., spleen, liver, and lung) and less in the TME. Importantly, the pH-dependent VISTA.18 demonstrated undiminished efficacy compared to VISTA.16 in combination with PD-1 blockade in the syngeneic MC38 tumor model [68]. No further information has been reported regarding plans for the clinical development of VISTA.18. 

SNS-101 is a fully human IgG1κ monoclonal antibody developed by Sensei Biotherapeutics that recently entered phase 1 clinical trials (NCT05864144). SNS-101 was developed in collaboration with Adimab Inc., leveraging a robust yeast-based surface display platform expressing large, fully human IgG libraries. Candidate mAbs were obtained through iterative selections for high-affinity VISTA binding at pH 6.0 versus binding at pH 7.4. Surface plasmon resonance analysis of the binding kinetics of SNS-101 demonstrated a greater than 600-fold selectivity for active VISTA at pH 6.0 (K_D_ = 0.218 nM) versus physiologic pH (K_D_ = 132 nM) [71]. As predicted, given the low affinity at pH 7.4, SNS-101 fails to bind to neutrophils and monocytes in whole blood, lacks TMDD, and demonstrated both dose-proportional drug concentrations and linear elimination kinetics in a single-dose PK study in cynomolgus macaques [71,72]. In addition, SNS-101 demonstrated significant reduction in cytokine release in multiple in vitro and in vivo assays compared to non-pH-dependent antibodies [71,72].

It could be argued that the likelihood of discovering pH-dependent, conditional blocking antibodies targeting the VISTA/PSGL-1 checkpoint was relatively high given the histidine-rich pH-dependent “switch domain” regulating the physiologic binding of these two proteins. Yet, considerable antibody screening and/or engineering was still required to achieve the high degree of pH-selectivity required to avoid TMDD and on-target/off-tumor activity. The generalizability of using large library screening to identify pH-mediated conditional antibodies remains to be determined.

**Generation of pH-dependent antibodies in the absence of histidine-rich target epitopes.** In 2011, the anti-CTLA-4 monoclonal ipilimumab became the first immune checkpoint mAb to be approved as a cancer agent. Since then, a critical limitation of ipilimumab use has been significant on-target/off-tumor activity manifesting as severe autoimmune adverse events, likely due to the inhibition of peripheral regulatory T cell function. Thus, conditionally active, tumor-selective anti-CTLA-4 antibodies would potentially display an improved therapeutic window. In contrast to VISTA, CTLA-4 binding to its receptors, CD80 and CD86, does not appear to be intrinsically regulated by pH. In fact, there is only a single histidine residue located in its extracellular domain. Despite this, two groups have published data on the generation of pH-dependent anti-CTLA-4 mAbs. 

In 2021, Chang et al. reported the discovery of a pH-dependent CTLA-4 mAb using the protein-associated chemical switches (PaCS) screening approach pioneered by BioAtla, Inc. [7]. In brief, the investigators generated a protein library of ipilimumab variants with mutations in the CDRs. These variants were then screened in ELISA assays in the presence or absence of physiologically relevant ions (the conceptual basis of PaCS), including sodium chloride, sodium bicarbonate, sodium sulfide, and lactic acid. Using their PaCS-based screening approach, a set of antibodies were identified with a range of pH-sensitivity. Clone 87CAB3 was identified as the lead antibody, demonstrating the greatest “binding selectivity”. At pH 7.4, the EC50 (ELISA) was reduced to 0.5% of the EC50 at pH 6.0. Clone 87CAB3 demonstrated equivalent anti-tumor efficacy as the parental CTLA-4 (ipilimumab analogue, “IpA”) in syngeneic MC38 tumors implanted into human CTLA-4 knock-in mice. The percentage of CD8 TIL and the CD8/Treg ratio was not significantly different between tumors treated with 87CAB3 or ipilimumab. However, the ipilimumab-treated mice exhibited an increase in percentage of CD4 effectors in the peripheral blood that was not seen in the mice treated with the low pH-selective clone. This result suggests that pH-dependent inhibition by clone 87CAB3 decreased the risk of on-target/off-tumor immune-mediated toxicity. The ability of the conditionally active 87CAB3 antibody to improve the toxicity profile of CTLA-4 blockade was subsequently tested in a non-human primate model using repeat administrations of anti-PD-1 (nivolumab analogue, “NiA”) combined with anti-CTLA-4 (IpA versus 87CAB3). In combination with anti-PD-1, both 87CAB3 and a second pH-dependent clone, 87CAB2, showed significantly decreased GI-related immunotoxicity compared to animals receiving IpA [7]. 

In addition to the primary focus on pH-dependent anti-CTLA-4 clones, Chang et al. [7] briefly mentions successful PaCS-based campaigns identifying conditionally active antibodies targeting EpCAM, Her2, Nectin4, CD73, and CD3. The authors argue that pH-dependent, conditionally active antibodies that are not necessarily contingent on histidine residues being present in the CDRs can be readily discovered. While they do not specify the sequence changes in 87CAB3 responsible for the high degree of selectivity for CTLA-4 at pH 6.0 versus 7.4, the authors note that the creation of low pH-selective mAbs derived from pH-independent prototype antibodies usually involves mutations where “[m]ostly polar and non-polar amino acids are replaced by charged amino acids”, with the most frequent being substitutions of aspartic acid, glutamic acid, and histidine [7]. This finding may arise from the unique features of the PaCS approach. Nevertheless, it is likely that in most cases, pH sensitivity will be most easily engineered by focusing on histidine content.

In independent work, Lee and colleagues used a structure-informed library design and phage-display screening to also generate pH-selective anti-CTLA-4 mAbs [8]. A series of histidine residues were substituted into the CDRs of ipilimumab in and around the paratope contacting CTLA-4. In addition, since light chain (LC) CDR1 binds in proximity to the single histidine residue in the extracellular domain of CTLA-4, targeted aspartic acid and glutamic acid substitutions were introduced into LC-CDR1. After several rounds of phage selection with binding at pH 6.0 and elution at pH 7.4 and further engineering to combine mutations found in different clones, a set of anti-CTLA-4 antibodies with a spectrum of pH-dependent binding characteristics were produced and analyzed in detail. In particular, two clones, Ipi.105 and Ipi.106, were selected for further evaluation based on their demonstrating selective binding to CTLA-4 at pH 6.0 versus pH 7.4 [8]. Ipi.106 was noted to have only minimal binding and antagonistic activity at neutral pH. The crystal structures of both antibodies were determined in complex with CTLA-4 to determine the structural basis of their pH-dependence. Both Ipi.105 and Ipi.106 employ the same five acidic amino acid substitutions (S31H; N55H; S27E; S30E; Y32E), whereas Ipi.106 has an additional heavy chain (HC) CDR3 T95H mutation. The S31H mutation common to both antibodies is found proximal to a group of negatively charged residues in CTLA-4 formed by E48, D64, and D65. Additionally, the N55H mutation is spatially near the E33 residue of CTLA-4. In the LC-CDR1, the S27E, S30E, and Y32E mutations comprise a group of negative charges that make contact with the single histidine residue in CTLA-4. Interestingly, the single additional mutation present in Ipi.106 (T95H in HC-CDR3) occurs near the center of the binding interface; the authors suggest that the presence of the bulkier side chain may account for the critical “de-tuning” of binding at neutral pH [8].

Both Ipi.105 and Ipi.106 were evaluated in human CTLA-4 knock-in mice implanted with MC38 tumors. Both pH-dependent antibodies resulted in anti-tumor activity and survival compared to Ipi, as well as similar levels of intratumoral Treg depletion. T cell activation in the draining lymph nodes was enhanced in mice treated with Ipi, whereas this increase was absent in mice treated with the pH-dependent antibodies, suggesting that systemic on-target/off-tumor effects had been successfully attenuated [8].

**Figure 2:**
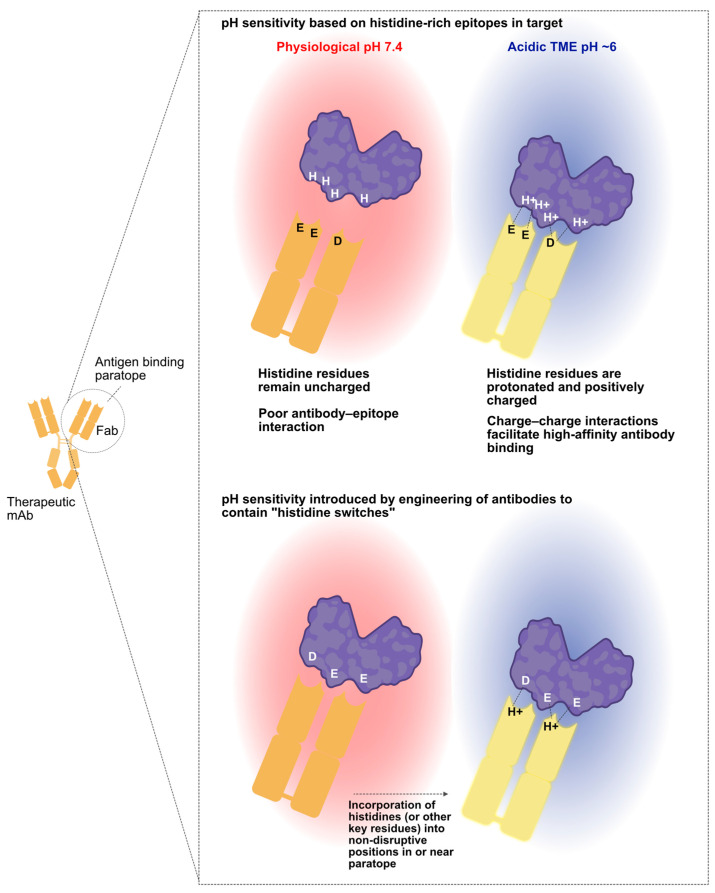
pH sensitivity can arise from either the target or the antibody. Some therapeutic targets will have histidine patches, enabling the identification of antibodies that preferentially bind at acidic pH when these histidine residues are protonated. Such interactions are primarily driven by electrostatic forces (**top**). If the therapeutic target lacks pH-sensitive surface epitopes, one may still identify conditionally binding antibodies by modifying a non-pH selective antibody. This involves introducing histidines or other changes into the antibody paratope, ensuring pH sensitivity is added while maintaining high-affinity target binding and de-tuning binding at physiological pH. Created in part with BioRender.com.

**The critical role of histidines.** The binding of an antibody to its target antigen is mediated through a constellation of non-covalent interactions between the epitope (binding site on the antigen) and paratope (binding site on the antibody), including van der Waals forces and electrostatic interactions between charged amino acid residues. Histidine is the only natural amino acid that converts from the neutral, deprotonated state to the charged, protonated state upon moving from physiologic pH (7.4) to the acidic pH ranges (<6.5) found in tumors [73]. Thus, this unique residue constitutes a critical pH-regulated “switch” (Figure 2). It is not surprising that nature utilizes histidine-driven pH sensitivity in regulating physiologic interactions such as that of VISTA–PSGL-1 binding or FcRn-mediated release of antibodies in the endosome. As such, the presence of histidine—in either the epitope or paratope—is likely a critical factor in achieving strong selectivity for low pH binding versus binding at physiologic pH. As demonstrated by the pH-dependent anti-VISTA and anti-CTLA-4 campaigns described above, the critical histidine residues may reside in the antigen, the antibody CDRs, or both. In the case of VISTA, multiple histidines are present in the PSGL-1 binding domain, which is the epitope targeted by both VISTA.18 and SNS-101. In the case of CTLA-4, it appears that electrostatic interaction with a single protonated histidine in the ECD at acidic pH is sufficient to establish pH selectivity. This suggests that rational histidine mutagenesis of CDRs may be a preferred strategy for improving the acidic pH selectivity of existing therapeutic monoclonal antibodies, while the large-scale, conditional library screening approach may be best suited for the development of novel candidate antibodies.

**Importance of de-tuning affinity at physiological pH.** To optimize the therapeutic window of a pH-dependent antibody, attention must be paid not just to selectivity (i.e., ratio of binding at acidic versus physiologic pH) but also to adequate “de-tuning” of binding affinity at physiologic pH, such that the antibody has very low target binding at pH 7.4. For example, a candidate antibody that had ~20-fold selectivity for binding at pH 6 versus 7.4 but retained low nanomolar binding of target at pH 7.4 would likely display on-target/off-tumor activity. Despite significantly improved binding at low pH, such an antibody may not be developable into a viable drug. The extent to which this is problematic will likely also depend on target expression levels in tumor versus non-tumor tissues.

**Structure-guided engineering versus agnostic screening.** In this review, we have highlighted several different strategies taken by investigators to develop conditionally active, pH-dependent antibodies selective for binding in the acidic tumor microenvironment. The approaches range from agnostic dual-pH selection of sequence-rich, fully human yeast surface display libraries (e.g., SNS-101) or PaSC-based selection of mutated CDR libraries derived from a non-pH dependent parent, to crystal structure-informed and directed histidine mutations. All of the above approaches have demonstrated success, with the caveat that the focused approach of improving the pH selectivity of a therapeutic antibody requires, of course, that the parent antibody has all of the desired features (e.g., correct target epitope and developability) and only lacks pH selectivity. Agnostic screening of sequence-rich, diverse antibody sequences has the advantage that antibody discovery is not contingent on the existence of a known parent antibody.

## 4. Conclusions and Future Directions

The expression of immune receptor targets on immune cells both in the tumor and in non-tumor tissue makes the problem of on-target/off-tumor activity particularly problematic in immuno-oncology. However, recent advances in antibody engineering and antibody surface display approaches have enabled groundbreaking progress in the identification and development of conditionally active antibodies that selectively bind their targets in the acidic milieu of the tumor microenvironment but remain relatively inert in normal tissues, where pH is tightly regulated at 7.4. One important exception noted earlier is that highly proliferating T cells shift into Warburg metabolism and lower the pH in microniches in lymph nodes and, presumably, other areas of lymph node activation [53]. An important corollary is that therapeutic antibodies with selective activity at acidic pH will potentially be active in immunologic sites such as tumor-draining lymph nodes [74]. For T cell checkpoint targets such as VISTA and CTLA-4, activity both in the immune synapse and in activated lymphoid microenvironments may represent a significant secondary locus of action that contributes to their anti-tumor activity.

As described above, the development of conditionally active, pH-dependent antibodies has been achieved by multiple groups employing different approaches. These initial successes support the general utility of the discovery process for pH-dependent mAbs, not just for immune-regulatory targets such as CTLA-4 and VISTA, but also for improving the on-target/off-tumor effects of targeting tumor-associated antigens on the cell surface of tumor cells. To our knowledge, the most clinically advanced of such programs are two BioAtla pH-dependent ADCs targeting AXL and ROR2, both of which are currently in phase 2 trials [75,76,77]. Other significant efforts have been made in this space, including the work by Sulea et al. developing conditionally active Her2 antibodies [78]. As this approach continues to gain traction, we expect that pH-dependent, conditionally active antibodies with selective activity in the tumor microenvironment will be developed to target other immune ligands, where on-target/off-tumor activity is a limitation. Strongly selective antibodies with stringently de-tuned binding at physiologic pH may improve the therapeutic window to such a degree as to enable their use in very potent antibody-derived modalities such as CAR-Ts, bispecific antibodies and bispecific T-cell engagers [79], ADCs, and radiopharmaceuticals. Based on the success of these initial programs reviewed here, we anticipate that conditionally active, pH-dependent antibody therapeutics will emerge as a potent new class of therapeutics in immuno-oncology.

## Data Availability

No new data were created or analyzed in this study. Data sharing is not applicable to this article.
